# Differential Gene Expression of Malaria Parasite in Response to Red Blood Cell-Specific Glycolytic Intermediate 2,3-Diphosphoglycerate (2,3-DPG)

**DOI:** 10.3390/ijms242316869

**Published:** 2023-11-28

**Authors:** Ana Balau, Daniel Sobral, Patrícia Abrantes, Inês Santos, Verónica Mixão, João Paulo Gomes, Sandra Antunes, Ana Paula Arez

**Affiliations:** 1 Global Health and Tropical Medicine (GHTM), Associate Laboratory in Translation and Innovation towards Global Health (LA-REAL), Instituto de Higiene e Medicina Tropical (IHMT), Universidade NOVA de Lisboa (UNL), 1349-008 Lisbon, Portugal; a21001549@ihmt.unl.pt (A.B.); patriciaabrantes@ihmt.unl.pt (P.A.); inesmariasantos2001@gmail.com (I.S.); santunes@ihmt.unl.pt (S.A.); 2 Genomics and Bioinformatics Unit, Department of Infectious Diseases, National Institute of Health Doutor Ricardo Jorge (INSA), 1649-016 Lisbon, Portugal; daniel.sobral@insa.min-saude.pt (D.S.); veronica.mixao@insa.min-saude.pt (V.M.); j.paulo.gomes@insa.min-saude.pt (J.P.G.); 3 Veterinary and Animal Research Centre (CECAV), Faculty of Veterinary Medicine, Lusófona University, 1749-024 Lisbon, Portugal

**Keywords:** *Plasmodium falciparum*, infection, erythrocyte, pyruvate kinase deficiency, enzymopathy, 2,3-bisphosphoglycerate, transcriptome, nanopore sequencing technology

## Abstract

Innovative strategies to control malaria are urgently needed. Exploring the interplay between *Plasmodium* sp. parasites and host red blood cells (RBCs) offers opportunities for novel antimalarial interventions. Pyruvate kinase deficiency (PKD), characterized by heightened 2,3-diphosphoglycerate (2,3-DPG) concentration, has been associated with protection against malaria. Elevated levels of 2,3-DPG, a specific mammalian metabolite, may hinder glycolysis, prompting us to hypothesize its potential contribution to PKD-mediated protection. We investigated the impact of the extracellular supplementation of 2,3-DPG on the *Plasmodium falciparum* intraerythrocytic developmental cycle in vitro. The results showed an inhibition of parasite growth, resulting from significantly fewer progeny from 2,3-DPG-treated parasites. We analyzed differential gene expression and the transcriptomic profile of *P. falciparum* trophozoites, from in vitro cultures subjected or not subjected to the action of 2,3-DPG, using Nanopore Sequencing Technology. The presence of 2,3-DPG in the culture medium was associated with the significant differential expression of 71 genes, mostly associated with the GO terms nucleic acid binding, transcription or monoatomic anion channel. Further, several genes related to cell cycle control were downregulated in treated parasites. These findings suggest that the presence of this RBC-specific glycolytic metabolite impacts the expression of genes transcribed during the parasite trophozoite stage and the number of merozoites released from individual schizonts, which supports the potential role of 2,3-DPG in the mechanism of protection against malaria by PKD.

## 1. Introduction

Malaria remains one of the biggest world public health problems, mainly affecting the poorest areas of the globe and contributing to poverty and inequality [1]. The last World Malaria Report, released in 2022, estimated that malaria has caused 247 million cases in 2021, from which approximately 619,000 have resulted in deaths [2]. The economic impact of malaria is also vast as it includes costs related to health care, decreased productivity and investment [3]. The emergence and spread of the resistance of mosquitoes to insecticides, and parasites to antimalarial medicines, highlight the urgent necessity for new, efficacious antimalarial tools with minimal adverse effects [4].

Human genetic traits that provide protection against malaria have been selected and have reached high frequencies in endemic areas [5]. Understanding the underlying mechanisms of this protection may unveil targets for new antimalarial approaches that could mimic the protective effects exhibited by naturally occurring red blood cell (RBC) disorders [6]. Such tools may allow the delay of life-threatening parasite densities until clearance through immunity or via a co-delivered antimalarial drug. Additionally, creating an unfavorable environment to the parasite by targeting host metabolic pathways may also help avoid drug resistance, as the therapeutic targets are not within, or produced by, the pathogen.

Pyruvate kinase deficiency (PKD) is an erythrocyte enzymopathy caused by mutations in the *pklr* gene and has been associated with resistance to malaria in murine models [7] and with reduced RBC infection in *P. falciparum* cultures [8]. Some of these mutations appear to have been positively selected in malaria-exposed populations [9,10,11,12] but the mechanisms underlying protection remain unknown.

Pyruvate kinase (PK) catalyzes the last step of glycolysis. PK-deficient RBCs display decreased ATP and pyruvate production and increased concentrations of 2,3-diphosphoglycerate (2,3-DPG) [13], synthesized on the RBC-specific Rapoport–Luebering shunt by bisphosphoglycerate mutase (BPGM). The parasite depends on glucose to obtain energy during blood-stage development, and it holds a complete set of glycolytic enzymes that seem to differ biochemically and structurally from their host counterparts, with the exception of BPGM [14]. In a situation of PKD, host PK activity sharply decreases, and 2,3-DPG content rises. Therefore, since *Plasmodium* parasites do not metabolize 2,3-DPG [14], we hypothesized that 2,3-DPG accumulation could have detrimental consequences for it by rendering an intracellular environment unsuitable for parasite development and providing a potential mechanism of protection against infection.

Previous results showed that addition of 2,3-DPG to an in vitro *P. falciparum* culture medium significantly impaired the parasite’s intraerythrocytic developmental cycle (IDC), the metabolic profile of 2,3-DPG-treated infected cells became more similar to that of non-infected cells, and host cells were not significantly affected. Also, parasites exposed to 2,3-DPG produced significantly fewer progeny, independently of the ATP level inside the cell [15,16]. Therefore, to confirm these preliminary results and understand the biological processes involved in this response, in this study, we analyzed the differential gene expression and the transcriptomic profile of schizogonic *P. falciparum* trophozoites from in vitro cultures subjected or not subjected to the action of 2,3-DPG, using Nanopore Sequencing Technology.

## 2. Results

### 2.1. Effect of 2,3-DPG on Parasite Intraerythrocytic Development

**Figure 1:**
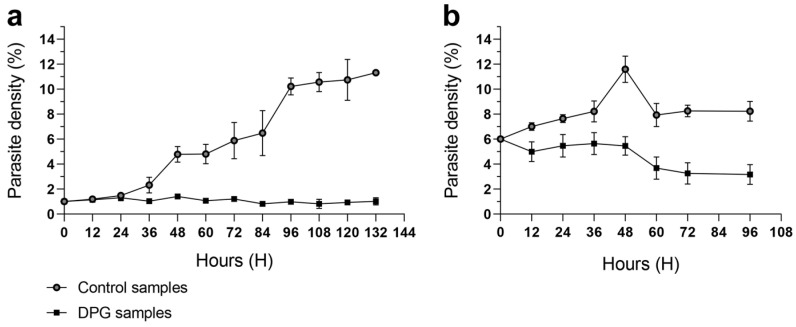
Parasite density (%) of *P. falciparum* 3D7 cultures that were untreated (control, light dots) or treated with 2,3-DPG 8 mM (dark squares). Assays were performed in triplicate for each condition using ring-stage synchronized cultures with initial parasite densities of 1% (**a**) and 6% (**b**). Error bars represent the standard deviation at each timepoint. First addition of 2,3-DPG 8 mM to the culture medium occurred at t = 0 h; growth was monitored until lysis.

2,3-DPG 8 mM was added to the culture medium for the first time at 0 h. In cultures with an initial parasite density of 1% (Figure 1a), untreated cultures exhibited a significant increase in parasite density until 96 h–108 h, reaching a plateau of 10% and 11%. In contrast, cultures treated with 2,3-DPG maintained a consistent parasite density of around 1% throughout the entire assay. This result is consistent with previous findings, and the same effect persisted in cultures with increased volumes and higher initial parasite densities (Figure 1b).

**Figure 2:**
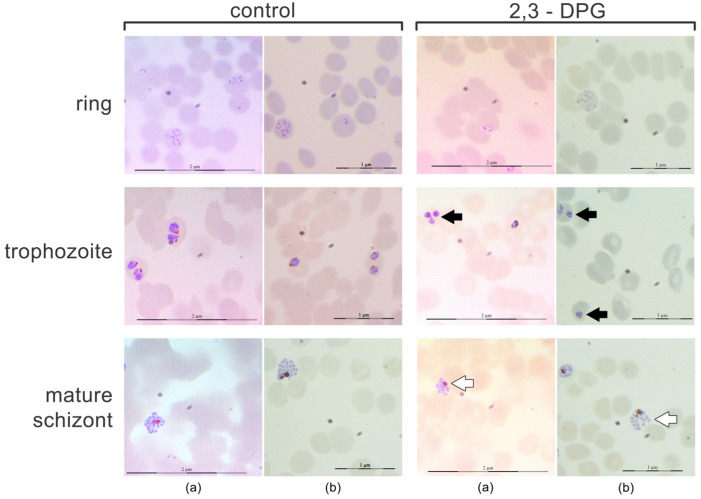
Morphological analysis of schizogonic stages—ring, trophozoite and schizont—every 12 h between 46 and 48 h of growth. Giemsa-stained smears (optical microscope 1000× magnification) of *P. falciparum* 3D7 cultures in the absence (control) and presence of 2,3-DPG 8 mM (2,3-DPG) and at 1% (a) and 6% (b) parasite densities. Less developed trophozoites (black arrows) and smaller schizonts and smaller progeny (lower number of merozoites) (white arrows) are visible in treated cultures.

A morphological analysis of each stage of the parasite—ring, trophozoite and schizont—was conducted using smears prepared every 12 h between 46 and 48 h of growth (Figure 2). The same trend was observed in cultures with both parasite densities of 1% and 6%. No gametocytes were observed in either of the cultures. At the ring stage, no discernible morphological differences were noted between parasites from treated and untreated cultures. However, trophozoites from cultures treated with 2,3-DPG exhibited a smaller size and appeared to possess higher cytoplasmic density. Similarly, differences were observed between treated and untreated mature schizonts, with those treated with 2,3-DPG displaying a reduced size and seeming to yield fewer progeny (lower number of merozoites produced per mature schizont).

**Figure 3:**
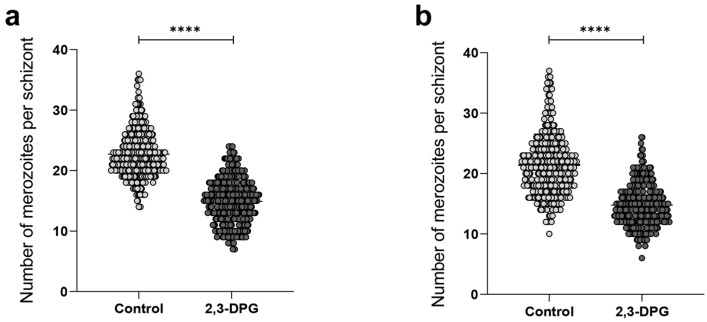
Number of merozoites produced by mature schizont in the absence (light dots) and presence of 2,3-DPG 8 mM (dark dots). in each triplicate of treated and untreated cultures with 1% (**a**) and 6% (**b**) parasite densities, 100 schizonts were considered. **** *p*-value < 0.0001. (**a**) Control: median = 22.00; mean = 22.73; SD = 4.07, and 2,3-DPG: median = 15.00; mean = 14.91; SD = 3.76. (**b**) Control: median = 21.00; mean = 21.42; SD = 5.12, and 2,3-DPG: median = 14.00; mean = 14.77; SD = 3.561.

To validate the impact of the compound 2,3-DPG on the parasites’ progeny, a count of merozoites produced by each mature schizont was performed every 12 h between 46 and 48 h of growth in each triplicate culture, totaling 100 schizonts per triplicate. The results derived from the merozoite count per schizont revealed a significant distinction between samples (*p*-value < 0.0001) (Figure 3). The number of merozoites generated by schizonts in cultures treated with 8 mM 2,3-DPG was notably lower compared to the number produced by schizonts in untreated cultures, applicable to both 1% and 6% parasite density cultures (Figure 3a,b, respectively). The higher count of merozoites per mature schizont did not surpass 26 in cultures treated with 2,3-DPG. Conversely, in untreated cultures, the maximum count of merozoites per mature schizont reached 37.

### 2.2. Differentially Expressed Genes between Treated and Untreated Cultures

For the assessment of differential gene expression between trophozoite-stage parasites (30 hours post-invasion (hpi)), exposed or not exposed to 2,3-DPG, three replicates of each condition were used for RNA-Seq analysis. A total of 2,303,056 reads were obtained from which 69.1% passed the quality check, corresponding of 1,590,847 reads with a median read length of 1422 bp, distributed across all libraries (barcodes). Details on the quality control (QC) of the sequencing data are provided in Appendix A.

Sequencing reads passing all the QC steps were aligned to the *P. falciparum* 3D7 reference genome. A total of 1,571,574 reads were mapped, corresponding to 98.8% of total pass reads with an average accuracy of 91.52% and an average quality between 19.1 and 20.3 (statistics and quality for the alignment and mapping of the reads of each library are available in Appendix A).

**Figure 4:**
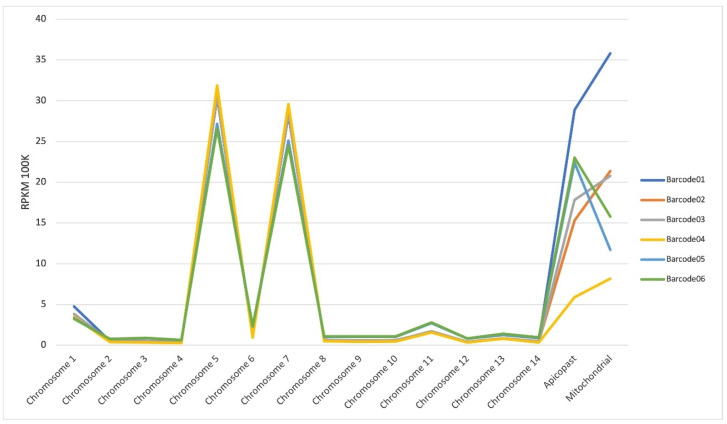
RPKM (reads per kilobase mapped) per chromosome. Control (untreated samples: barcode01, barcode02, barcode03); DPG (treated samples: barcode04, barcode05, barcode06).

The mapped reads across all 14 chromosomes and apicoplast and mitochondrial genomes of *P. falciparum* are shown in Figure 4. Our results show that chromosomes 1, 5, 7 and 11 exhibited a higher number of mapped reads, both for untreated (barcodes 01, 02 and 03) and treated samples (barcodes 04, 05 and 06). Most of these mapped reads correspond to RNA ribosomal genes (PF3D7_0112300, PF3D7_0112700, PF3D7_0531600, PF3D7_0531800, PF3D7_0532000, PF3D7_0725600, PF3D7_0725800, PF3D7_0726000, PF3D7_1148600, PF3D7_1148620 and PF3D7_1148640) or histones (PF3D7_1105000 and PF3D7_1105100). The same occurred regarding the apicoplast and mitochondrial genomes, with a higher number of mapped reads corresponding to the genes PF3D7_API04900, PF3D7_API05700, PF3D7_API05900, PF3D7_API06700 and PF3D7_MIT02100.

**Figure 5:**
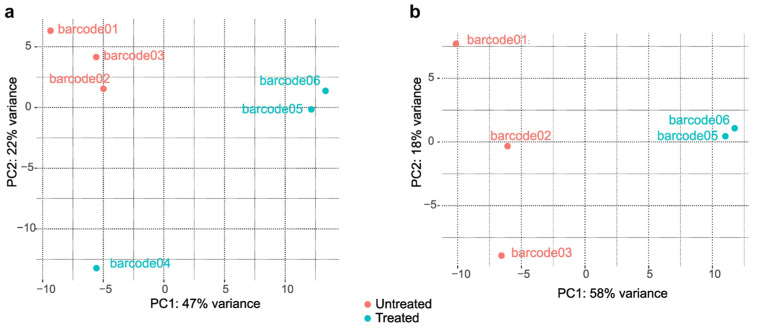
Principal component analysis (PCA) of the normalized counts for each barcoded library, with and without barcode04 ((**a**) and (**b**), respectively).

Principal component analysis (PCA) of the normalized counts for each barcode revealed an effect of treatment, with barcodes 05 and 06 clustering together, as well as the three replicates of untreated cultures (barcodes 01, 02 and 03), with PC1 and PC2 explaining 47% and 22% of the variance, respectively (Figure 5a). However, barcode04 displayed distinct behavior from both groups, possibly due to technical issues during sample preparation. Subsequent analysis was conducted excluding reads originating from this library (Figure 5b). In this revised analysis, PC1 effectively separated treated samples from the untreated samples, accounting for 58% of the variance. Tabular files containing data with and without these reads are available in the Supplementary Materials—Tables S1 and S2, respectively.

**Figure 6:**
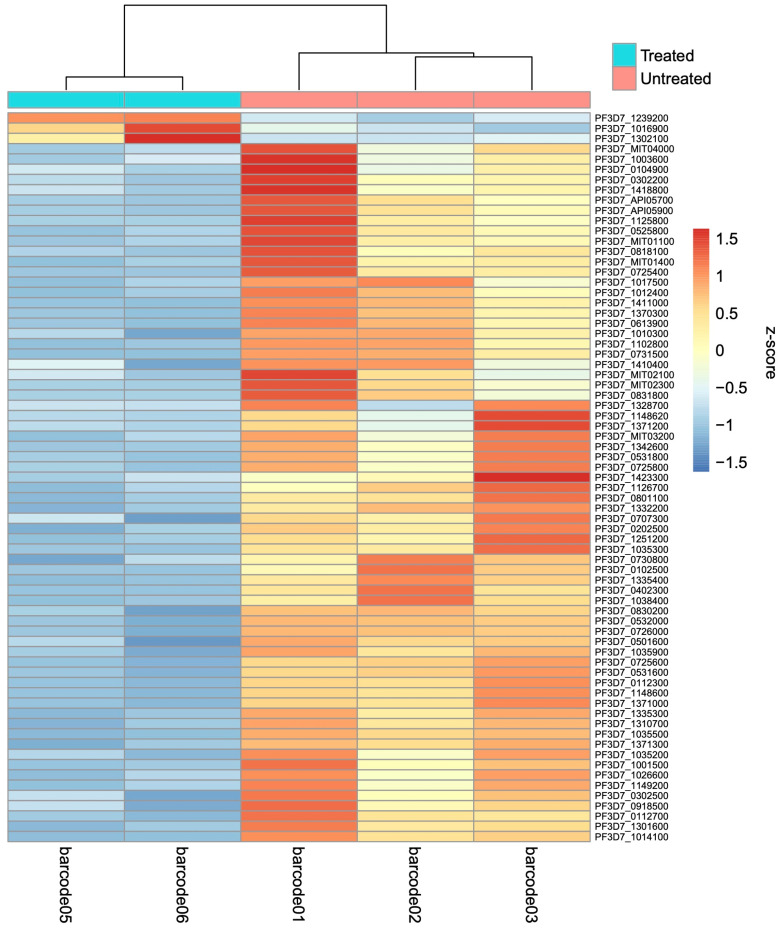
Heatmap of the differentially expressed genes between treated (barcodes 05 and 06) and untreated samples (barcodes 01, 02 and 03).

A differential gene expression analysis between the two conditions was performed and revealed a total of 71 genes that were differentially expressed, two genes from the apicoplast genome (PF3D7_API05900 and PF3D7_API05700), six from the mitochondrial genome (PF3D7_MIT04000, PF3D7_MIT03200, PF3D7_MIT01400, PF3D7_MIT02300, PF3D7_MIT02100 and PF3D7_MIT01100) and 63 genes from the nuclear genome. From the total number of genes that were differentially expressed, three genes (4.2%) were upregulated (PF3D7_1302100, PF3D7_1239200 and PF3D7_1016900) and 68 genes (95.8%) were downregulated in the cultures subjected to 2,3-DPG (Figure 6 and Supplementary Table S3).

A heatmap plot displays the five barcoded samples on the *X*-axis that cluster based on treatment, and the 71 differentially expressed genes on the *Y*-axis. Replicates from the treated samples (barcode05 and barcode06) show very similar levels of gene expression, and the same is observed for untreated samples (barcode01, barcode02 and barcode03), but substantial differences in gene expression levels are observed between the two conditions, which indicate that the presence of 8 mM 2,3-DPG in the culture medium caused a variation in the expression of genes transcribed during the trophozoite stage (30 hpi).

#### Gene Ontology Enrichment Analysis

To characterize the differentially expressed genes, we performed GO assignments for the three main GO categories: molecular function (MF), biological process (BP) and cellular component (CC). GO analysis showed that the most significant enriched GO terms were GO:0008201~heparin binding in the MF category, GO:0044409~entry into the host in the BP category and the GO term GO:0020008~rhoptry in the CC category (Table 1; gene products are detailed in Table S3).

Upon analyzing the metabolic pathways where the differentially expressed genes may be involved (Table S3), we observed that a total of 16 genes are annotated as coding proteins involved in cell cycle control, most of them downregulated. Among these, PF3D7_1423300 (serine/threonine protein phosphatase 7) is annotated as a protein involved in steps during passage through prophase, and PF3D7_0525800 (inner membrane complex protein 1g, putative) is annotated as a protein predicted to be involved in the cell cycle regulatory network and control of microtubule assembly. Also involved in the cell cycle regulatory network are PF3D7_1038400 (gametocyte-specific protein) and PF3D7_1239200 (AP2 domain transcription factor, putative), the latter being upregulated. The other 12 genes are annotated as functional orthologs of known cell cycle proteins in *Escherichia coli*.

Two of the downregulated genes are related to the effect of hyperoxia on gene expression (PF3D7_1125800 (Kelch domain-containing protein, putative), PF3D7_1017500 (myosin essential light chain ELC)).

Three genes have been found in previous studies to be differentially expressed in severe malaria (PF3D7_0613900 (myosin E, putative), PF3D7_0202500 (early transcribed membrane protein 2) and PF3D7_1102800 (early transcribed membrane protein 11.2)), significantly correlated with coma score (PF3D7_1335400 (reticulocyte binding protein 2 homologue a)), and 10 genes have been considered as candidate genes related to virulence.

The three protein-coding genes in the mitochondrial genome of *P. falciparum* (PF3D7_MIT01400-cytochrome c oxidase subunit 3, PF3D7_MIT02300-cytochrome b and PF3D7_MIT02100-cytochrome c oxidase subunit 1) are shown to be downregulated in samples subjected to 2,3-DPG.

## 3. Discussion

*Plasmodium* has coevolved with humans for thousands of years, shaping both the parasite and its human host. Interactions between the host and infectious agents play a pivotal role in determining individual susceptibility and the morbidity experienced by infected individuals. Our research efforts have been dedicated to exploring the significance of PKD in conferring resistance against malaria.

We postulated that the accumulation of 2,3-DPG resulting from PKD might lead to an intracellular environment that is unfavorable for the *Plasmodium* parasite during its infection of RBCs, thus contributing to the mechanism of protection against malaria. Apart from the inhibitory effect on the glycolytic pathway upstream and the pentose phosphate shunt [17,18], as well as low intracellular ATP and pyruvate levels [13], elevated concentrations of intracellular 2,3-DPG also result in a decrease in pH in order to maintain Donnan equilibrium [19]. In fact, other RBC disorders already associated with protection against malaria infection, such as beta thalassemia, G6PD deficiency or sickle cell disease [20], also imply an increase in 2,3-DPG levels due to the activation of glycolysis upstream of pyruvate kinase [21,22,23], and we cannot exclude that this endogenous host metabolite may also be involved in these protective mechanisms.

To investigate the interaction between the parasite and its host cell, we conducted an analysis using in vitro *P. falciparum* cultures with the addition of 2,3-DPG to the culture medium. Ideally, RBCs from individuals with PKD should be employed; however, obtaining the necessary amounts of blood from these anemic individuals is challenging. Therefore, we validated the suitability of the model by observing a similar effect on the parasite, achieved either by introducing 2,3-DPG into the culture medium or inducing a PKD phenotype through enolase inhibition [15].

In the present study, we confirmed previous findings that when exposed to the influence of 2,3-DPG, parasites were unable to normally proceed to a new cycle of growth. In untreated cultures, an exponential rise in parasite density is evident, aligned with the 48 h life cycle of the parasite. During this cycle, the schizont ruptures, releasing up to 32 merozoites [24], poised to invade new erythrocytes. However, this elevation in parasite density did not manifest in cultures treated with 2,3-DPG. The parasite could develop in the compound’s presence between 0 and 24 h, exhibiting identical parasite densities in untreated and treated cultures, but it failed in progression by augmenting its parasite density in successive cycles. Earlier studies have also demonstrated that 2,3-DPG’s impact on RBC deformability and membrane zeta potential was relatively minor, and these changes did not impede effective parasite re-invasion or egress. Moreover, cytotoxicity remained low, with no discernible alterations in ATP levels and no noticeable effect on the parasite when RBCs were subjected to 2,3-DPG treatment before infection [15,16]. We hypothesize that a distinct entry mechanism for exogenous 2,3-DPG in infected and non-infected RBCs may occur. This is likely due to the increased permeability of the infected cells’ membranes resulting from parasite metabolic activity [25,26], which needs to be confirmed in future studies. These findings strongly imply a direct influence of 2,3-DPG on the parasite.

The inhibition of parasite growth is likely attributed to an impediment in parasite maturation, consequently yielding notably reduced progeny from parasites subjected to the treatment. Regulation of progeny number may arise due to nutrient exhaustion or stress factors [27,28]. However, it can also be impacted by intrinsic or extrinsic host factors, as replicated in the current study. Consequently, to delve into the biological mechanisms driving this phenomenon, we examined the impact of 2,3-DPG on parasite gene expression.

We analyzed the differential gene expression between *P. falciparum* trophozoites from in vitro cultures subjected or not subjected to the action of 2,3-DPG. At present, only a few malaria studies based on nanopore technology have been reported addressing DNA sequencing diagnostics and genotyping [29,30,31], the de novo assembly of *Plasmodium* sp. genomes [32,33] or the analysis of transcript isoforms [34]. To our knowledge none have addressed differential expression under different environmental conditions.

The presence of 2,3-DPG in the culture medium caused a clear effect on the expression of genes transcribed during the trophozoite stage at 30 hpi, which is the approximate timepoint when the first round of DNA replication begins in *P. falciparum* asexual blood stages [35]. The parasite then undergoes several rounds of DNA replication and nuclear division that are not directly followed by cytokinesis, resulting in multinucleated cells. The number of generated nuclei in *Plasmodium* directly predicts the number of progeny emerging from an infected cell [28]. Our results show that several genes differentially expressed, most of them downregulated, are related to cell cycle proteins that control the progression of a eukaryotic cell through the various phases of the cell cycle, which may explain the observation of reduced progeny from treated parasites.

In addition to the lower progeny, morphological analysis revealed less developed trophozoites and smaller schizonts when parasites were treated with 2,3-DPG. In infected RBCs, glucose uptake and lactate production increase almost 100-fold [36], and a disruption of the parasite glycolytic chain could affect parasite development or proliferation [37]. At least at the transcription level, no impairment of parasite glycolysis occurred, since we could not detect significant differences in the expression of the genes that code for key enzymes of glycolysis, such as hexokinase (PF3D7_0624000), phosphofructokinase (PF3D7_1128300), pyruvate kinase (PF3D7_0626800), and phosphoglycerate kinase (PF3D7_0922500), in untreated and treated samples. However, the three protein-coding genes in the mitochondrial genome were downregulated, and this may impact the energy production in the parasite’s mitochondria, affecting its survival and replication. Furthermore, an effect related to the effect of hyperoxia on gene expression was observed. It is known that hyperoxia may influence the parasite’s metabolic pathways and affect its growth and reproduction [38,39]. 2,3-DPG is an allosteric regulator of oxygen affinity to hemoglobin, and increased 2,3-DPG levels inside the cell would result in decreased O_2_-bound hemoglobin and a variation in O_2_ pressure, leading to direct exposure of the parasite to hyperoxia.

The transcriptionally active genome varies during the IDC [40]. The present analysis focused on the trophozoite stage (30 hpi), the most metabolically and transcriptionally active asexual stage [41]. We observed enrichment in the GO terms associated with nucleic acid binding, transcription and monoatomic anion channel activity, consistent with biological processes that are known to occur at this stage, such as translation and DNA replication and active metabolism [41,42]. However, strong enrichment was also observed in processes related to entry into the host cell, rhoptry and microneme, the reason for which is not clear. Not only are these processes predominant in schizonts, we have not observed before any differences in the invasion of RBCs by treated or untreated parasites [15,16].

Although the effect of treatment with 2,3-DPG is clearly shown, this study has some limitations. Poly (A) selection may inconsistently bias mRNA expression [43] and the low number of replicates per condition and high variability within replicates, especially regarding the untreated samples, prevent definitive conclusions. We consider the present study as preliminary, but it has important exploratory value as a pilot to further studies with a larger number of replicates to complement the work described in this paper. However, we show that the analysis based on Oxford Nanopore Technology (ONT) serves as an effective approach to detecting the effects of different treatments on parasites. Although the major limitation of nanopore sequencing is its lower read accuracy when compared with short-read technologies, the advantages of long reads outweigh low reads’ accuracy [44]. ONT long-read sequencing provides real-time, amplification-free, single-molecule sequencing of cDNAs that enables the recovery of full-length transcripts without the need for an assembly step, and so, quantifying the expression of genes can be achieved via simple counting of the assigned reads [34,45,46].

To have the comprehensive picture of the effect of 2,3-DPG or the PKD on the *Plasmodium* parasite, future studies should address the full IDC, validate confirmed differentially expressed genes via quantitative RT-PCR and perform subsequent functional analysis. Also, the possibility of reading full-length transcripts, and thus, efficiently identifying RNA molecules and transcript isoforms, including transcript length and splice isoforms, opens up perspectives for the analysis of regulatory roles and splicing events triggered by the exposure of a parasite to different intracellular conditions [34,47,48].

## 4. Materials and Methods

### 4.1. Blood Donors

Healthy type 0 RBCs were collected from adult volunteer donors (*n* = 5). Whole venous blood was collected in 1 g/dL EDTA tubes (Sarstedt, Nümbrecht, Germany) and immediately washed four times with PBS pH 7.0 (Sigma-Aldrich, Dramstadt, Germany) to remove plasma and buffy coat. RBCs were left at 50% hematocrit in complete Gibco Roswell Park Memorial Institute 1640 Medium (cRPMI (1.044% (*w*/*v*) Biowest, Nuaillé, France) supplemented with 0.59% (*w*/*v*) HEPES (Sigma-Aldrich, Dramstadt, Germany), 0.005% (*w*/*v*) hypoxanthine (Sigma-Aldrich, Dramstadt, Germany), 0.5% (*w*/*v*) AlbuMAXII (Gibco, Thermo Fisher Scientific, Eindhoven, The Netherlands) and 0.2% (*w*/*v*) sodium bicarbonate (Merck, Darmstadt, Germany), buffered to pH 7.0 to 7.2) and stored at 4 °C for no longer than three weeks to ensure no major changes in ATP and 2,3-DPG levels [49].

Blood variants of genes associated with malaria protection that could influence parasite growth were ruled out through the molecular diagnosis of the polymorphisms most common in Portugal, namely, of the genes HBB—hemoglobin subunit beta; *pklr*—pyruvate kinase, liver and red blood cell; and *g6pd*—glucose-6-phosphate dehydrogenase, as described by Morais et al. [15]. Every donor was wild-type for all studied genes.

All donors were clearly informed that participation in the study was voluntary and were made aware of the objectives of the work. Informed consent form was obtained from each participant before blood collection, and a numerical code was assigned to each donor to maintain confidentiality.

### 4.2. Plasmodium falciparum In Vitro Cultures

*Plasmodium falciparum* 3D7 parasites (BEI Resources MRA-102) were maintained in RBCs at 5% hematocrit at 37 °C in a wet atmosphere with 5% CO_2_, accompanied by daily cRPMI changes [50]. Parasite growth was monitored daily through estimation of the parasite density (percentage of infected cells) in 20% Giemsa-stained (Giemsa’s Azur-eosin–methylene blue, Sigma-Aldrich, Darmstadt, Germany) thin blood smears.

All assays were performed after synchronization of cultures with 5% (*w*/*v*) sorbitol (Sigma-Aldrich, Dramstadt, Germany), following an adapted protocol [51], and initiated with ring-stage forms 6–8 h post-invasion (hpi).

### 4.3. Effect of 2,3-DPG on Parasite Intraerythrocytic Development

To study the effect of the compound on parasites, solutions of 2,3-diphospho-D-glyceric acid pentasodium salt (Sigma-Aldrich, Darmstadt, Germany) 1.33 M were prepared in ultrapure water, from which intermediate dilutions were carried out with cRPMI. The synthetic compound 2,3-DPG was added to the culture medium at a concentration of 8 mM as it has previously been shown that this concentration impairs the in vitro parasite growth cycle by 50% after 48 h of treatment [15].

A culture of ring-stage parasitized RBCs was divided into 12 new cultures with 5% hematocrit and 1% and 6% parasite densities (six each). To six cultures, we added 8 mM 2,3-DPG, and the remaining six cultures were not subjected to the treatment. Daily changes of medium, supplemented or not supplemented with 2,3-DPG, were performed, and no RBCs were added to the cultures throughout the assay.

After 12 h of growth, 6 µL of each culture was collected in triplicate in a 96-well flat-bottom plate with a lid, and the parasite density was read using a flow cytometer (CytoFLEX, Beckman Coulter, Brea, CA, USA). A total of 100 µL of 0.5× SYBR Green solution in PBS and 94 µL of RPMI medium were added to each well, and plates were incubated for 45 min at 37 °C and 5% CO_2_. After centrifugation, cells were washed and resuspended in the same volume of PBS. Three independent assays were performed in triplicate. This procedure was repeated every 12 h until the lysis of the cultures. Circa 1000 RBCs were analyzed per well, and the parasite density was calculated using FlowJo v10 software (Tree Start Inc., Ashland, OR, USA).

Thin blood smears were made every 12 h between 46 and 48 h of growth and observed via light microscopy (Olympus BX40, 1000×, Tokyo, Japan) for the morphological analysis of parasites; images were captured using the ProgRes Capture Pro 2.1 program. In the same smears, the number of resulting merozoites from nuclear division in mature schizonts (segmentation stage) were counted. In total, 100 mature schizonts per replicate, from treated and untreated cultures, with both 1% and 6% parasite densities, were considered.

### 4.4. RNA Preparation

To obtain a minimum RNA amount of 100 ng, recommended for cDNA library preparation, it was necessary to elevate the parasite density. Therefore, total parasite RNA was obtained from six new synchronized cultures of *P. falciparum* with 5% hematocrit and parasite density ranging from 7.5% to 11%. A total of 8 mM 2,3-DPG was added to three cultures, and the remaining three cultures were not subjected to the treatment. Cultures were maintained in standard conditions and the parasite was isolated for 30 h of the first growth cycle, corresponding to the stage of trophozoite (confirmed via optical microscopy). From each culture, the parasite was isolated from 300 µL of parasitized RBCs with 1 mL of 0.05% saponin (Sigma-Aldrich, Dramstadt, Germany) and incubated on ice for five minutes according to the procedure described by Lee et al. [34].

Afterwards, total parasite RNA was extracted from the three replicates of both treated and untreated synchronized cultures of *P. falciparum* at the trophozoite stage (30 hpi) using NZYol reagent, and processed according to the manufacturer’s instructions (Nzytech, Lisbon, Portugal). Integrity and quantity of RNA were determined using a Qubit 4.0 fluorometer (Invitrogen, Eindhoven, The Netherlands) and a NanoDrop 1000 spectrophotometer (Thermo Fisher Scientific, Eindhoven, The Netherlands).

### 4.5. cDNA Library Construction and Transcriptome Sequencing

For the construction of cDNA libraries, Direct cDNA Sequencing kits (SQK-DCS109) and Native Barcoding Expansion 1–12 (EXP-NBD104) from Oxford Nanopore Technology (ONT, Oxford, UK) were used according to the manufacturer’s protocol. The latter allows for the multiplex and simultaneous analysis of up to 12 different samples subjected to different treatments.

cDNA libraries were constructed from the triplicate cultures for each condition (supplemented or not supplemented with 2,3-DPG). Briefly, reverse transcription was followed by enzymatic digestion of the RNA template and synthesis of the second strand of cDNA. At the end, the poly-A ends were repaired, and the barcode sequences and the sequencing adapters were ligated. Six barcodes were ligated to each sample (untreated samples: barcode01, barcode02 and barcode03; treated samples: barcode04, barcode05 and barcode06).

Barcoded libraries were quantified using a Qubit fluorometer, by employing a Qubit 1x dsDNA HS assay kit (Invitrogen, Eindhoven, The Netherlands). Barcoded libraries were pooled together, loaded on a MinION R9.4.1 flow cell (ONT, Oxford, UK) and sequenced at MinION Mk1C (ONT, Oxford, UK) for 24 h (until the number of viable nanopores significantly reduced). Sequences were obtained through MinKNOW software v1.10 onboard MinION, and only “pass reads” were used in the analyses.

### 4.6. Analysis of Gene Expression

Base calling was performed using Guppy v5.1.13 software from MinKNOW v4.5.4, and FastQ files were generated with reads separated per barcode in folders. Each barcode folder was concatenated in a simple file with a Unix command line, and quality was checked using pycoQC v2.5.2 and FastQC v0.74 on the platform Galaxy and Fastq Control Experiment v3.7.3 from Epi2Me Desktop Agent (ONT, Oxford, UK).

The absence of barcode sequences was verified in each concatenated file using a Unix command line, through the command gunzip -c “name_of_the_file”.fastq.gz|grep 0: “barcode_sequencing”|wc −1. Minimap2 v2.24 was used to align and map the reads to the *P. falciparum* 3D7 reference genome (source version GCA_000002765.3), downloaded from PlasmoDB r.63. SAMtools stats v2.0.4 and Artemis Software Sanger r.18.2.0 were used to check mapping quality. The numbers of mapped reads to each of the 14 chromosomes of *P. falciparum* were checked using Samtools IdxStats v2.0.4 and MultiQC v1.11. The program featureCounts v2.0.3 was used to count mapped reads assigned to each gene match in an annotation file, after selecting the “Long reads” option. The annotation file was obtained from PlasmoDB r.63. Differences in gene expression were analyzed through DESeq2 v2.11.40.7. Genes were considered differentially expressed under the effect of 2,3-DPG (treated/untreated) when the logarithmic (base 2) difference in expression was different from zero and a corrected *p*-value of less than 5% (*p* < 0.05) was obtained (log_2_(FC) > 0 indicates upregulation and log_2_(FC) < 0 indicates downregulation of genes). Graphical representation of differentially expressed genes was performed using Heatmap2 (v3.1.3), as described in the Reference-based RNA-Seq data analysis workflow [52]. Gene Ontology (GO) enrichment analysis was carried out using gene sets based on a corrected *p*-value < 0.05 with GO enrichment v2.0.0.

### 4.7. Statistical Analysis

Statistical analysis related to the effect of 2,3-DPG on the parasite was performed using the program GraphPad Prism v8 (GraphPad Software). Comparison of merozoite count per mature schizont was performed using the unpaired Student’s *t* test method. The statistical significance level was set at *p* < 0.05.

## 5. Conclusions

In this study, we confirmed previous results that state that the addition of 2,3-DPG to an in vitro *P. falciparum* culture medium significantly impaired parasites’ IDC, due to a significant reduction in the progeny produced by treated parasites, and we show that this may be due to the downregulation of several genes related to cell cycle control.

The number of merozoites released from an individual schizont is a key determining factor for the multiplication rate of parasites. Lower multiplication rates would benefit the majority of individuals early in infection, due to the delayed onset of high parasite densities and slower progression to anemia, potentially preventing the development of severe malaria early in infection. Host mechanisms such as systemic host inflammation [53] or enzymopathies such as PKD, and associated metabolites, may trigger transcriptional alterations in circulating blood-stage *Plasmodium* trophozoites and may impair parasite maturation in vivo. Research must continue to assess whether these mechanisms may provide adjunct tools to enhance the effectiveness of existing antimalarial drugs.

## Figures and Tables

**Table 1:** Summary of the most significant enriched GO terms according to the Gene Ontology enrichment analysis of the 71 genes differentially expressed in trophozoite-stage parasites at 30 hpi, exposed to 2,3-DPG.

GO Category	GO Term	Name	N. of Genes	Study Freq.	Pop. Freq.	*p*-Value	*q*-Value
Molecular Function	GO:0008201	heparin binding	6	25%	0.68%	1.48 × 10^−9^	8.11 × 10^−8^
	GO:0005488	binding	23	96%	64%	2.70 × 10^−4^	2.97 × 10^−3^
	GO:0005253	monoatomic anion channel activity	2	8.3%	0.15%	3.15 × 10^−4^	2.97 × 10^−3^
	GO:0046812	host cell surface binding	6	25%	4.4%	4.36 × 10^−4^	3.00 × 10^−3^
Biological Process	GO:0044409	entry into host	11	50%	7.4%	7.87 × 10^−8^	9.05 × 10^−6^
Cellular Component	GO:0020008	rhoptry	7	18%	1.3%	3.22 × 10^−7^	1.87 × 10^−5^
	GO:1903561	extracellular vesicle	9	24%	2.9%	8.73 × 10^−7^	2.53 × 10^−5^
	GO:0016459	myosin complex	2	5.3%	0.10%	4.35 × 10^−4^	4.2 × 10^−3^
	GO:0020039	pellicle	2	5.3%	0.10%	4.35 × 10^−4^	4.2 × 10^−3^
	GO:0070258	inner membrane pellicle complex	5	13%	2.0%	8.80 × 10^−4^	6.38 × 10^−3^
	GO:0033643	host cell part	15	39%	18%	1.67 × 10^−3^	9.89 × 10^−3^
	GO:0009986	cell surface	6	16%	4.6%	7.31 × 10^−3^	0.0353
	GO:0020009	microneme	3	7.9%	1.2%	9.88 × 10^−3^	0.0441

Study Freq.—number of genes within the 71 genes differentially expressed associated with the GO term; Pop. Freq.—the number of genes in the entire background population (5427 genes) associated with the GO term.

**Figure A1:**
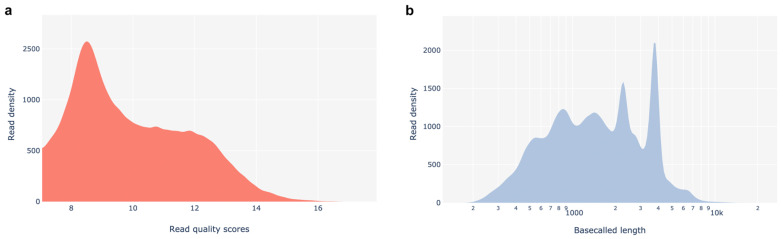
Summary of the quality control of sequencing data (according to PycoQC). (**a**) Base-called reads’ PHRED quality—distribution of the Qscores for each read giving a global quality score for each read. (**b**) Base-called reads’ length—minimum read length around 200 bp and maximum read length around 10,000 bp.

## Data Availability

The RNA sequencing data presented in this study are openly available in the Sequence Read Archive (SRA) of the National Center for Biotechnology Information (NCBI) of the National Institute of Health (NIH), USA, with accession number PRJNA1036731. Additional resulting data sets are included in the article as well as the Supplementary Files.

## Supplementary Materials

The supporting information can be downloaded at: https://www.mdpi.com/article/10.3390/ijms242316869/s1.

## Appendix A

### Appendix A.1. Transcriptome Sequencing Analysis—Quality Control

#### Appendix A.1.1. RNA Quality and cDNA Library Sequencing

The RNA concentrations ranged from 60.4 to 412.0 ng/µL in a final volume of 20 µL, with integrity values between 5.5 and 10.0. The purity, as assessed using the A260/A280 ratio, varied within an optimal range of 2.04 to 2.22. The final concentration of barcoded libraries was 7.14 ng/µL, corresponding to 85.68 ng of cDNA in a final volume of 12 μL.

Barcoded libraries were pooled together and sequenced on a MinION R9.4.1 flow cell uing a MinION Mk1C device for 24 h, yielding a total of approximately 2.3 million reads. Quality control confirmed the absence of barcode sequences. According to pycoQC software v2.5.2, sequences with a Qscore equal to or higher than 7 were considered ‘pass’ reads. From the total number of reads analyzed (2,303,056 reads), 69.1% of reads passed the FastQ quality check, corresponding of 1,590,847 reads with a median read length of 1422 bp, distributed across all libraries (712,209 were unclassified or poor-quality sequences). The number of pass reads and sequence length per barcode are represented in Table A1.

**Table A1:** Number of pass reads and sequence length per barcode.

	Control (Untreated Samples)			DPG (Treated Samples)		
	Barcode01	Barcode02	Barcode03	Barcode04	Barcode05	Barcode06
Number of Pass reads (%)	124,497 (7.83%)	262,680 (16.51%)	165,905 (10.43%)	246,274 (15.48%)	225,089 (14.15%)	566,402 (35.60%)
	553,082 (34.77%)			1,037,765 (65.23%)		
Total number of bases (Mbp)	201.3	423.2	270.9	537.4	414.6	1000.0
Sequence length	128–46,300	117–23,141	110–21,960	104–29,694	141–29,207	127–30,804

The base-called reads’ PHRED quality plot obtained using PycoQC indicates that most reads have Qscores between 8 and 12, centered around 8.44, and show a median PHRED score of 9.57. The distribution of pass read lengths shows that the minimum read length is around 200 bp, while the maximum read length reaches approximately 10,000 bp. The plot displays a dispersed distribution, and about 10% of pass reads exceed 3 kb in length (Figure A1).

#### Appendix A.1.2. Alignment and Mapping of Reads against the 3D7 Reference Genome

Reads from all the libraries were aligned and mapped against the *P. falciparum* 3D7 reference genome. Statistics for the mapped reads, including the proportion of mismatches per mapped bases (error rate) and mapping quality, are presented in Table A2. A total of 1,571,574 reads were mapped, corresponding to 98.8% of total pass reads, with an average accuracy of 91.52% and an average quality between 19.1 and 20.3.

**Table A2:** Statistics and quality of the alignment and mapping of the reads of each library against the *P. falciparum* 3D7 reference genome (source version GCA_000002765.3).

		Total N. of Sequences	Mapped Reads	Unmapped Reads	Error Rate	Average Length	Average Quality
**Control** (untreated samples)	Barcode01	124,497	122,745 (98.6%)	1752 (1.4%)	9.63 × 10^−2^	1617	19.2
	Barcode02	262,680	257,908 (98.2%)	4772 (1.8%)	9.65 × 10^−2^	1611	19.1
	Barcode03	165,905	163,915 (98.8%)	1990 (1.2%)	9.63 × 10^−2^	1633	19.3
**DPG** (treated samples)	Barcode04	246,274	242,957 (98.7%)	3317 (1.3%)	9.49 × 10^−2^	2182	19.1
	Barcode05	225,089	222,821 (99%)	2268 (1%)	1.04 × 10^−1^	1842	19.8
	Barcode06	566,402	561,228 (99.1%)	5174 (0.9%)	1.07 × 10^−1^	1826	20.3
